# Teachers’ perceptions on including learners with barriers to learning in South African inclusive education system

**DOI:** 10.4102/ajod.v14i0.1543

**Published:** 2025-05-31

**Authors:** Jacomina M.C. Motitswe

**Affiliations:** 1 Department of Inclusive Education, College of Education, University of South Africa, Pretoria, South Africa

**Keywords:** barriers to learning, diverse learning needs, exclusion, inclusive education, participation

## Abstract

**Background:**

Inclusive Education acknowledges that all children can learn, but requires support. However, addressing learning barriers and responding to diverse needs remains a challenge in some South African schools, leading to the exclusion of some learners.

**Objectives:**

This study explored teachers’ perceptions of including learners who experience barriers to learning and responding to their diverse learning needs.

**Method:**

A qualitative case study was conducted using purposive sampling to select six schools across two geographical contexts within one district in the North West province, South Africa. Focus groups were conducted with six school-based support teams (three to five members each), and semi-structured interviews were conducted with six school principals.

**Results:**

Teachers expressed concerns about inadequate and limited training in inclusive education, which contributes to persistent negative attitudes. The continued application of the medical model still prevailed. Systemic challenges such as overcrowded classrooms, limited teaching and learning time, insufficient policy guidance, and inadequate support from district-based support teams were also highlighted.

**Conclusion:**

Teachers’ reluctance to implement inclusive education policies may be linked to perceptions of inadequate training and lack of resources to address diverse learner needs. Many teachers still follow the medical model rather than an inclusive approach to equitable education. A shift towards inclusive practices requires regular review and support to prevent learner exclusion.

**Contribution:**

The study contributes to policy and practice by advocating for ongoing review and enhancement of inclusive education strategies and pedagogies.

## Introduction

While inclusive education (IE) is globally embraced as a pathway to respond to the plea for equal education for all learners, especially those who experience barriers to learning, it is not yet in practice in some schools in South Africa (Engelbrecht 2020; Engelbrecht et al. 2015; Majoko et al. 2018). In principle, IE has much to offer in the sense that it focuses on maximising the participation of all learners in the cultures and curricula of educational institutions and, subsequently, minimising barriers to learning and development. Moreover, IE responds to the objective of the education system with regards to ensuring quality education for all learners, which would provide them with opportunities for lifelong learning, entering the world of work and ensuring meaningful participation in society as productive citizens (Engelbrecht 2020). Inclusion in education is about ensuring that every learner feels valued and respected and can enjoy a clear sense of belonging (Schuelka 2018).

In South Africa, the implementation of IE has been a central focus following the Salamanca Statement and the subsequent policies that advocate for the inclusion of learners with diverse needs in public ordinary schools (Engelbrecht 2020; Engelbrecht & Muthukrishna 2019). South Africa’s commitment to IE was formalised with the establishment of the Education White Paper 6 (EWP 6): Special Needs Education-Building and Inclusive Education and Training System in 2001, which laid the foundation for the transformation of the educational system to accommodate all learners (Department of Education [DoE] 2001; Engelbrecht & Muthukrishna 2019).

Many African countries have been devoted to the implementation of IE, with the aim of achieving education for all by addressing the issue of diversity and equality (Kinuthia 2022). In a South African context, much has been done regarding the development of material for learning and teaching support, curriculum development, human resources and the Screening, Identification, Assessment, and Support (SIAS) strategies (Department of Basic Education [DBE] 2014; Engelbrecht 2020). It, however, remains critical to determine whether policy has been translated into action and, if so, to what extent this has been done.

In undertaking our study, the authors assumed that many teachers have continued to implement IE policy in ways other than what was envisioned when publishing EWP 6 (DoE 2001). The mismatch between policy as it was intended and how it is implemented in practice may be based on teachers’ limited understanding of the policy and the context in which they find themselves (Kinuthia 2022). Furthermore, we concur with the findings of Engelbrecht (2020), Kinuthia (2022) and Singal and Muthukrishna (2014) that the challenges associated with the implementation of IE may be caused by the discrepancies in the cultural, social, financial and historical background of the education system. For example, in South Africa, the discrepancies include financial constraints that affect the availability of resources, attitudinal barriers caused by a lack of cultural acceptance of learners who experience diverse educational needs, and the beliefs and practices of teachers who believe in traditional teaching methods that are not conducive to inclusivity (Engelbrecht & Muthukrishna 2019; Singal & Muthukrishna 2014).

Inclusive education policies advocate for the full participation of learners with diverse educational needs. However, the reality on the ground often contradicts these ideals. Many learners who experience barriers to learning face systemic exclusion because of factors such as inadequate teacher training, limited resources and a lack of institutional support (Mpu & Adu 2021). As key implementers of IE, teachers play a vital role in promoting or hindering the inclusion process. While there is extensive research on implementing IE in South Africa, there is a lack of literature specifically addressing teachers’ perceptions in the North West province regarding the inclusion of learners who experience barriers to learning and how to meet their diverse learning needs. Understanding their perceptions and experiences is essential to identifying the barriers that perpetuate exclusion within an IE system and developing practical interventions to address them.

Our study thus focused on the following two questions:

- *What are the teachers’ perceptions of including learners who experience barriers to learning?*
- *What do teachers do to ensure that inclusivity in education is implemented?*

## Research methods and design

A qualitative multiple-case study approach was employed in our study to explore teachers’ perceptions of the inclusion of learners with barriers to learning.

A multiple case study design was adopted, aligning with Yin’s (2017) characterisation of multiple case studies as investigations involving two or more cases with both shared and distinct characteristics. Furthermore, Merriam and Tisdell (2016) highlight that a multiple case study approach explores real-life, bounded systems by engaging in comprehensive and detailed data collection from various sources. This design was selected to explore teachers’ experiences and perspectives on the inclusion of learners with barriers to learning, providing insights from a range of educational contexts.

### Sampling

Our study was undertaken in the Bojanala district in the North West province, South Africa. The North West province was one of four provinces that participated in a study by the Danish International Development Agency (DANIDA) that was funded to expand IE programmes; the Bojanala district was part of the pilot study (DoE 2002). The report indicates that most teachers from the Bojanala district were funded to further their studies in IE in collaboration with Wits University (Johannesburg), the University of North-West (Potchefstroom) and the Catholic Institute of Education (Johannesburg) (DoE 2002). For the purposes of our study, further information and advice were sought from the Bojanala DBST regarding the selection of schools. Purposive sampling was found to be appropriate (Yin 2017). Six schools were selected: two mainstream primary, two full-service and two special schools. One full-service school, which was the first school to be selected as a full-service school to pilot the implementation of IE was selected because the authors assumed that the teachers at the school were already familiar with inclusive practices and knew the gaps or challenges. One special school that was selected was the first to be converted into a special school as a resource centre in the Bojanala district. The two mainstream schools were selected because some of the teachers were funded by DANIDA during the pilot project. All the schools selected were situated in two different geographical contexts (Madibeng and Rustenburg circuits; see Table 1).

**TABLE 1:** Description of schools.

School	Resource centre	District	Circuit
A	Special school as resource centre	Bojanala	Madibeng
B	Full-service	Bojanala	Madibeng
C	Full-service	Bojanala	Rustenburg
D	Mainstream	Bojanala	Rustenburg
E	Mainstream	Bojanala	Madibeng
F	Special school	Bojanala	Rustenburg

### Data collection methods

Two data collection methods were used, namely focus group discussions with school-based support teams (*n* = 6 groups of three to five members, respectively) and semi-structured individual interviews with school principals (*n* = 6) (see Table 2 and Table 3, respectively.) The questions intended to solicit information on teachers’ perceptions of including learners who experience barriers to learning, meeting diverse learning needs in the classroom and involving the learners in learning. Questions further prompted teachers’ understanding of IE and how they support learners who experience barriers to learning.

**TABLE 2:** Participants in the focus group (*N* = 24).

School	Total number of SBST members	Acronyms for participants
A	4	FGA 1, FGA 2, FGA3, FGA4
B	5	FGB 1, FGB 2, FGB 3, FGB 4, FGB 5
C	4	FGC 1, FGC2, FGC3, FGC 4
D	4	FGD1, FGD2, FGD3, FGD4
E	3	FGE 1, FGE2, FGE3
F	4	FGF 1, FGF 2, FGF 3, FGF4

SBST, school-based support team; FGA, focus group from school A; FGB, focus group from school B; FGC, focus group from school C; FGD, focus group from school D; FGE, focus group from school E.

**TABLE 3:** Participants in the individual semi-structured interviews (*N* = 6).

School	Individual interview: Principal	Acronym for principal
A	1	PA
B	1	PB
C	1	PC
D	1	PD
E	1	PE
F	1	PF

PA, principal from school A; PB, principal from school B; PC, principal from school C; PD, principal from school D; PE, principal from school E; PF, principal from school F.

The semi-structured interview schedule used the same questions as the focus groups but also included a range of questions to capture a detailed picture of the principals’ perceptions of the implementation of IE in schools. The questions focused on the challenges experienced when implementing IE, teachers’ developmental needs and the strategies used for teacher empowerment at the schools. The interviews and focus group discussions were recorded using a voice recorder.

### Data analysis

Data processing steps consisted of preparing and organising the data, selecting keywords and quotations, coding the data into categories, searching for themes and reporting the findings (Naeem et al. 2023; Xu & Zammit 2020).

### Ethical considerations

All ethical obligations were adhered to for the purposes of ours study. Firstly, ethics approval was sought from the College of Education Ethics Review Committee at the University of South Africa (Unisa) (No. 2015/10/14/30074703/01/MC). Secondly, permission letters were obtained from the North-West Department of Education and the principals of the six identified schools. Confidentiality was also preserved by using pseudonyms to identify the participants.

## Results

The findings highlighted three themes that contribute mostly to the inclusion and/or exclusion of learners and deal with diverse learning needs. These themes emerged from data collected through focus groups and semi-structured individual interviews. The themes included the medical deficit model of teaching, learning and support; inclusion or exclusion of learners who experience barriers to learning; and policy implementation. Table 4 provides a synopsis on the themes and sub-themes that emerged. Furthermore, the evidence of the findings is provided in verbatim quotations from the focus groups and interviews.

**TABLE 4:** Themes emerged from data.

Theme	Sub-themes
The medical deficit model of teaching, learning and support	Teachers’ experiences and understanding of inclusive education Lack of support for learners who experience barriers to learning Limited time Lack of parental support
Inclusion and/or exclusion of learners who experience barriers to learning	Withdrawal of learners from the regular classroom or remedial classes Labelling learners
Policy implementation	Curriculum Assessment Policy Statement (CAPS) Admission policy

### Theme 1: The medical deficit model of teaching, learning and support

Several participants displayed a negative attitude and frustration about addressing and responding to diverse learner needs in their classrooms. They seemed to be uncertain about how to support learners who experience barriers to learning and were frustrated about the poor level of support received from the DBE district officials. Participants from all six schools identified systemic challenges as having a direct and distinct effect on their daily teaching and learning activities, including teachers’ understanding of the concept of IE, limited available support to learners who experience barriers to learning and limited support to teachers themselves. One of the participants shared the following view during a focus group:

‘You see the problem is, teachers take inclusive education as a monster. It confuses them a lot, and they don’t even want to hear about it…. It is difficult to provide support whereas we need that support as well.’ (FGC3)

The participant’s response corroborates what Engelbrecht and Muthukrishna (2019) mention, that teachers’ attitudes towards IE and their understanding of its meaning influence the success of its implementation. The participant’s statement reflects a pervasive anxiety and resistance among teachers towards IE, which they describe as a ‘monster’ that causes confusion and frustration. This metaphor suggests a serious concern and lack of clarity surrounding IE, highlighting both emotional and professional challenges faced by teachers. Such reluctance can undermine the effectiveness of inclusive practices, as successful implementation relies on teachers’ willingness and ability to embrace and adapt to inclusive principles (Mfuthwana & Dreyer 2018). However, some of the participants made suggestions on how to improve such negative experiences during focus groups. Some of the suggestions are captured in the following contributions:

‘As a full-service school, my suggestion is that if there can be a remedial class where these learners with barriers to learning can be referred to. The school should also be provided with a psychologist to assist these learners and to advise us as teachers on how we can address the barriers in our classrooms. We also don’t have relevant assistive devises to support our learners.’ (FGC2)

‘Some of the learning needs, want a specialist, this is just an example, psychology or special needs education, to be able to assist those learners with special needs. We are really not trained to address special needs, we are only trained on the teaching methodologies.’ (FGB4)

The participants’ statements highlight a perception that learners with barriers to learning require specialised intervention beyond what they can provide, suggesting adherence to the medical model of disability. This model conceptualises barriers to learning and disabilities as conditions requiring expert diagnosis and treatment, often resulting in the separation of learners with barriers to learning from mainstream classrooms (Kapp 2019). Such a perspective aligns with a belief that only specialists, such as psychologists or teachers trained in special needs, are equipped to support these learners effectively. This belief implies that mainstream teachers are inadequately prepared to address diverse learning needs within inclusive settings (Masuku et al. 2021).

In addition to the suggestions raised by participants in the focus groups, participants from both mainstream and full-service schools noted other systemic factors as negatively affecting the implementation of IE, such as overcrowded classrooms, limited time to provide support, especially in the intermediate phase, lack of support services and resources and feeling overworked. The following are participant’s views on the matter:

‘It is challenging because those learners have different barriers, so a learner who has barriers to learning, which requires individual attention cannot be given that attention fully because we have 50 – 60 learners in each classroom. If you give individual attention to one learner, the others are suffering. It’s really difficult for us to teach learners with different learning needs in one classroom which is overcrowded.’ (FGE1)

Additionally, participant FGE3 stated that the difficulty of overcoming barriers to learning is made worse by the teaching of multiple subjects. This is the comment:

‘I mean if you sit with a class of 55 learners, and also teaching more than one subject …. you still have to prepare for the next day for three different levels of abilities for all the subjects you are teaching.’ (FGE3)

The participants’ responses emphasise significant contextual challenges that impede the support of learners with barriers to learning in inclusive classrooms. The mention of classrooms with 50–60 learners points to issues of overcrowding, which limits teachers’ capacity to provide individualised attention. In such instances, learners who need additional support are often left unattended, as teachers want to balance the needs of an entire classroom. This reality reflects broader systemic barriers, where the teacher-learner ratio fails to accommodate diverse learning needs, thereby compromising the efficacy of IE (Mpu & Adu 2021). Furthermore, the burden of teaching more than one subject, each with varying levels of learner abilities, intensifies the strain on teachers. As indicated by the second participant (FGE3), preparing for multiple subjects across different ability levels within a single classroom becomes an overwhelming task.

Contrary to what the participants expressed above, there were positive practices from special schools. These responses sum up what the participants have raised:

‘To be honest, we did not experience any form of negativity or negative attitudes on addressing barriers to learning and diverse learning needs at our school. In actual fact, every teacher in this school is willing to provide assistance to all learners. They are really dedicated and committed to their work.’ (PA)

‘Teachers, therapists, learner support teachers, psychologists and nurses are always available to support our learners, so I think our kids here are very fortunate because they have all the support they need, so I think that is maybe where the special schools still have a critical role to play.’ (FGA1)

The responses from the participants confirm the positive practices observed in the special schools, justifying the commitment made by the DBE (2014) to provide support services to special schools, enabling them to offer more intensive support to learners with disabilities. The participants demonstrate how a well-resourced, collaborative and dedicated teaching environment can significantly impact learners with diverse needs.

In terms of limited time to support learners who experience barriers to learning, this experience was primarily voiced by participants from mainstream and full-service schools. They referred to limited time in class and to the unavailability of learners after school hours as primary concerns, stating that:

‘We don’t get enough time to assist our learners who have learning problems. Immediately when the period ends, the next teacher is already available to start teaching, so those learners who experience barriers to learning did not grasp anything when you were teaching, so they are always left behind.’ (FGB3)

‘Most of our learners are using transport to travel to school, so when the schools knock off, the transports are already here and learners go straight to them, so we cannot create extra time to assist our learners or to do remedial work.’ (FGE2)

The issues raised by the participants from mainstream and full-service schools on limited time serve as a barrier to effective support. Participants indicated that they must often move swiftly from one lesson to the next without the flexibility to provide additional support for learners with barriers to learning. Moreover, the participants revealed that learners’ reliance on scheduled transportation prevented them from staying after school for remedial support or additional assistance.

In contrast to what has been said, there are teachers who are trying their best to provide support. One of the participants said the following:

‘I always try to use the little time that I get to assist those learners who experience barriers to learning, like sometimes I create extra time during their break or after school, because we at the foundation phase, our day end at 13:30, so from 13:30 until 14:30.’ (FGC1)

Participants, including school principals, expressed a common concern regarding the limited support they receive from district officials. They highlighted the lack of guidance, resources and assistance for learners who have been identified as needing specialised support. Here are the participants’ views on the matter:

‘I’m discouraged by the lack of support from the side of the Department of Education, I mean the department just declare schools as full-service, but they don’t even provide support or resources to them, we are always on our own. We don’t get support at all from them.’ (FGC1)

In addition to what FGC1 mentioned, another participant raised the following:

‘Yes, we are the SBST, but truly speaking those district officials, or should I say the people high up there don’t give us support at all, in most cases we are on our own. I can say we are the SBST but cannot say we are functional because we don’t get support, we were not trained on our roles, we just read from the EWP 6 and other documents on our roles, no one is assisting us. So, I would say the challenge we experience is lack of support from the DBST.’ (FGD4)

The participants’ responses reflect frustration and helplessness, indicating a gap between policy intent and actual support provided to schools. Another participant noted that despite identifying learners with barriers to learning, particularly those who require high-level support, the DBST did not intervene. This lack of response underscores a systemic issue, where schools may not receive the support they need to provide appropriate interventions. The participants voiced the following:

‘We are faced with learners who need to be referred to special institutions and we informed the DBST and requested their intervention, but they have never come. We just keep them in the school, there is nothing that we can do….’ (FGD1)

In addition to the sub-themes, there was another serious concern regarding the lack of parental involvement; the participants thought that it also contributed to barriers to learning. The participants indicated the lack of sufficient parental involvement as a challenge for effectively supporting learners who experience barriers to learning, mentioning that parents may even contribute to the barriers to learning experienced by their children. A participant explained this view by stating that:

‘Most of learners’ parents are not supportive, because the learners will tell us that the parents don’t want to assist them, so you can see that the parents don’t motivate or encourage their children on their schoolwork.’ (FGC3)

Another participant revealed a threatening response they received from some parents:

‘One parent told us that what is happening in his family is none of our business, we work at school not in their homes, so we should teach the learners and stop enquiring about their family background, that is why we stopped visiting parents, otherwise we could end up being in trouble or being hurt.’ (FGD1)

Another participant provided another reason for limited parental involvement:

‘Some parents are on a denial state, they don’t want to accept that their children are experiencing barriers to learning.’ (FGC3)

The feedback from participants suggests that insufficient parental involvement limits the school’s ability to support learners effectively and, in some cases, worsens the challenges these learners face. The disconnect between home and school leads to a lack of comprehensive support for students, highlighting ongoing discrepancies in South Africa related to social issues and a lack of cultural acceptance for learners with diverse educational needs (Engelbrecht & Muthukrishna 2019).

### Theme 2: Inclusion and/or exclusion of learners who experience barriers to learning

The second theme, also consisting of sub-themes, was on inclusion and exclusion of learners with barriers to learning. Key issues that emerged from this theme include the negative impact of remedial class, labelling of learners and persistent tension within the school community regarding the inclusion of diverse learners.

The participants did not seem positive about learners being removed from class to attend remedial sessions, because of reasons captured in the following contribution:

‘I think it’s a problem to refer learners to the remedial class during lessons. It means they are separated from the class to the remedial class. Other teachers are willing to take over from where the remedial teacher has ended. However, most teachers are not willing to do that, they just continue with their work without thinking for the poor learners who were not in class during that period of time. That means the learners will be left behind each and every time they attend the remedial class.’ (FGB1)

For the participants, labelling learners presented specific challenges and did not benefit either the learners or the teachers who included these learners in their classes. Some participants expressed frustration over being labelled as ‘inclusive teachers’ by their colleagues. They also highlighted the various levels of challenges that learners may experience, emphasising the need for effective support. The responses show that most teachers still believe in the medical model, as stated by Masuku et al. (2021).

These sentiments are reflected in the following quotations:

‘Other teachers would refer their learners with barriers to our classrooms while standing on the corridors or outside the classroom, like talking loudly to us saying ‘because you have inclusive learners you can be able to help “those”… teachers in this school like to label learners, they even label us, imagine calling me inclusive teacher or inclusive learners. The teachers don’t even bother to tell us the problems that the learner have [sic] … Just imagine, how unprofessional it is.’ (FGE3)

‘What I noticed in our learners, it’s not that they cannot write, some are not committed to learn, they just write so that they can submit whatever activity given, they are actually very lazy, they don’t take efforts in their work.’ (FGE1)

Additionally, one participating principal expressed the following:

‘Remember this concept full-service school does not start now; it has been there even during our time. We have to attend with boys and girls who were not ready for school and the DoE does not have any place for these kids. You understand, so they must learn in a normal class whereas they are “not normal” for that matter.’ (P3)

The participant’s responses reflect that the issues of remedial, labelling, deficit perceptions and persistent biases reflect an education system that is struggling to transition from medical models to inclusive practices. Furthermore, the principal commented:

‘… I heard the Minister of Education talking about it this year. To my view and understanding, inclusive education and addressing barriers to learning at mainstream and full-service schools is still theory. The minister also said that this is still theory; the practical part of it is not possible.’ (P3-FSS)

Based on these remarks, it points to an entrenched cultural and historical misunderstanding of IE (Kinuthia 2022; Singal & Muthukrishna 2014). Contrary to what was raised, another principal was positive that IE can be implemented effectively. The participant concurred with Mfuthwana and Dreyer (2018) that attitudinal transformation can contribute to the successful implementation of IE.

‘If we can change our attitudes and mind shift, we can be able to implement inclusive education effectively.’ (P2)

Another point that was raised by one SBST member who is also a member of the school management team was on evaluating learners for admission.

‘This is a special school for [learners with] physical disabilities. It is a special school and resource centre. So, we do evaluate the learners before admit them. We cannot admit a learner with multiple disabilities as we are using the same curriculum that is used by mainstream schools. The admission team is the one that evaluate the learner. It consists of a psychologist, speech therapist, occupational therapists, and some members of the SBST team. If a learner displays multiple disabilities, or any intellectual or sensory disability, then we advise the parent to take the learner to a relevant institution that specialises with [sic] other multiple disabilities.’ (FGA1)

This feedback highlights significant challenges in the IE framework, especially regarding the effectiveness of the support structure. The response reveals several issues and contradictions within the current approach to IE, especially regarding the criteria for admission and the scope of services offered by special schools. It contradicts the DBE’s vision for special schools, which advocates for inclusive spaces that provide a specialised education programme to learners requiring access to highly intensive educational and other support, either on a full-time or a part-time basis (DBE 2014).

### Theme 3: Policy implementation

The participants cited several reasons why the implementation of policy documents did not align with their original intentions. Among these reasons were limited training opportunities and a lack of involvement in the policy development process. Additionally, participants noted that the national school curriculum (Curriculum Assessment Policy Statements [CAPS]) presented certain limitations in addressing barriers to learning. The following excerpts highlight the participants’ perspectives:

‘I think from the implementation of the EWP 6, there are many gaps one can talk about. Firstly, let us look at the SIAS policy … Teachers need to be thoroughly trained on it before even implementing it.’ (P3)

The participants’ concerns regarding the CAPS point to systemic issues, particularly regarding insufficient training on policies. Another point that was raised by one SBST member, who is also a member of the school management team, was about evaluating learners for admission. Referring to the CAPS document, some participants identified that CAPS has some flaws that make it inflexible.

‘The curriculum does not cater for learner pace, it does not consider learners who experience barriers to learning, and they have to complete activities within a specific period.’ (FGD3)

This sentiment illustrates that the way CAPS has been standardised may marginalise learners with barriers to learning who are unable to keep up with its pace. Another participant noted that CAPS falls short in offering instructional approaches that address diverse learning needs, stating that: ‘The CAPS only provides for the contents to be taught and assessment, it does not guide on addressing barriers to learning and responding to diverse learning needs.’ (FGE5)

In addition, the participants from mainstream and full-service schools indicated that they were always frustrated by the district officials, particularly those responsible for curriculum and assessment. They expressed their frustration towards district officials for not incorporating strategies to address learning barriers, which points to a lack of holistic and differentiated approaches necessary for supporting diverse learners. The following excerpts highlight the participants’ perspectives:

‘Those who are responsible for curriculum and assessment do not integrate addressing barriers to learning during their workshops, and they do not consider the fact that there were those learners who experience barriers to learning who need support.’ (FGC3)

‘Subject advisors focused on what should be taught, and the number of assessment tasks that should be done per term and they also expect common activities from all learners.’ (FGD3)

A further aspect that was raised by participants from the mainstream and full-service schools is the lack of intervention strategies. They raised the lack of accountability within the teachers. Participant FGC4 criticised the practice of recording intervention activities only towards the end of the year, implying that some teachers approached intervention as a compliance measure rather than a meaningful process to support learners. They voiced the following:

‘We should actually start with intervention process [sic] immediately when we have identified the learners, and it should first start with the class teacher, but you know what is done here? They only do interventions record towards the end of the year when we are supposed to submit the progression schedules to the district office. And they would point fingers at learners, forgetting that they also contributed in creating barriers to learning for those learners.’ (FGC4)

In addition, another participant noted that teachers often neglect to complete the necessary forms, and instead of conducting meaningful interventions, they progress learners who are not adequately prepared for the next grade level. This is the participant’s comment:

‘You see the other thing is, even teachers themselves, we give them the forms to guide them during the intervention process, but they do not complete them, so they just progress the learners even if they don’t deserve to progress to the next grade. Teachers do not want to account for learners who experience learning difficulties, they don’t even want a lot of paperwork.’ (FGE3)

According to the data collected, it is evident that there is an exclusion within IE systems. The findings also confirm that there is a lack of intervention strategies to support learners who experience barriers to learning.

## Discussion

The findings on exclusion within an IE system reveal that systemic barriers were experienced when responding to diverse learning needs and when implementing IE practices. These include the use of the medical deficit model of teaching, learning and support; inadequate policy implementation; and a lack of support to both teachers and learners who experience barriers to learning. The participants first expressed their attitude on the implementation of IE. This perspective is similar to the findings of Engelbrecht and Muthukrishna (2019), who argue that teachers’ attitudes and their comprehension of IE critically shape its implementation. When teachers view IE as a complex and overwhelming concept, they may feel inadequately prepared and unsupported, creating a cycle of unmet needs. Notably, the participants also pointed to the need for support among teachers themselves, revealing a potential gap in professional development and institutional resources required to empower teachers to manage diverse classroom needs effectively.

The challenge, therefore, lies in addressing both the practical and emotional support teachers require to facilitate IE successfully. This involves not only enhancing their understanding of IE but also fostering a supportive environment where teachers receive ongoing guidance and resources. Without such support, resistance may persist, impeding the development of inclusive classrooms where all learners can thrive.

Furthermore, the participants also raised some suggestions which they thought would address the barriers to learning; however, they were more reliant on the medical model. As argued by Masuku et al. (2021), the implication of the medical model perpetuates the practice of diagnosing, labelling and isolating learners with disabilities and those with barriers to learning, thereby excluding them from regular educational activities. This approach can hinder the IE goals of fostering acceptance, participation and equity for all students. Embracing a more inclusive model would involve capacitating mainstream teachers with strategies to support diverse learning needs, allowing all learners to thrive within an inclusive educational environment without unnecessary separation.

One of the findings was the ambiguity of the policy, advocating for a specialised type of education in some instances. For example, there are special schools that provide a more intensive to higher level of support compared to full-service schools that tend to provide a moderate to high level of support. These schools are said to receive support from the provincial and district DoE (2001, DBE 2014). Mainstream schools, however, provide mild to moderate support services. These schools are mostly left on their own and receive very limited support from district officials. Our findings justify what EWP 6 and the SIAS policies explain in terms of support services being rendered at the three types of schools (DBE 2014; DoE 2001). Teachers mentioned a lack of understanding of what constitutes IE, as it is a complex concept; hence, the confusion in its implementation (Donohue & Bornman 2014; Kinuthia 2022; Nel et al. 2014).

They also raised concerns about the shortfalls of CAPS, stating that it is inflexible and does not offer instructional approaches that address diverse barriers to learning. While policy documents like EWP 6, SIAS and CAPS aim to promote IE, their practical effectiveness is hindered by issues such as attitudes, teacher preparedness, curriculum inflexibility and a lack of support (Mpu & Adu 2021).

The teachers believed that learners who experience barriers to learning should be referred to specialised institutions. This confirms that the medical deficit model still plays a role. For example, it has been revealed that some teachers still label learners as intellectually challenged or special needs learners. These teachers still believe in remediation, where learners who experience barriers to learning are withdrawn from their classrooms to receive support separately from their peers. The practice of removing learners from mainstream classrooms to attend remedial sessions is a recurring concern. As expressed by participants from both mainstream and full-service schools, removing learners separates them from the primary learning environment, which may inadvertently label them as ‘different’ and ‘needing help’ in ways that can lead to social exclusion (Haegele & Hodge 2016; Kapp 2019).

While remedial classes are intended to provide support to learners with barriers to learning, they can inadvertently put these learners at risk and reinforce marginalisation. This occurs when learners miss critical components of the curriculum, which increases the likelihood that they may fall further behind in their education.

It has also been discovered that evaluation is used for admission at a special school as a resource centre, which, according to the EWP 6 and SIAS policies, provides high-level support (DBE 2014; DoE 2001). According to Slee (2019) and Walton (2018), exclusion in education is a process of denying learners access to participation in the education system, particularly at schools. Therefore, the findings revealed by participants from the special school as a resource centre justify that exclusion is implemented.

Another systemic barrier comprises the need for more resources and adequate support from the district officials, especially for mainstream schools. Support is an important aspect of meeting diverse learning needs and reducing barriers to learning. Therefore, the aim is to ensure learning access and participation for all learners to reach their potential (DBE 2014). Participants from full-service and mainstream schools indicated that they did not receive adequate support from the DBST, which hindered their ability to assist learners experiencing barriers to learning. Furthermore, the participants from mainstream schools stated that the DBST were more committed to the special schools and full-service schools. However, two participants from the full-service school in the Rustenburg circuit indicated that they had learners who experienced severe barriers to learning and were identified for interventions by the DBST; however, the team had not intervened or given a recommendation for those learners to be referred to relevant educational institutions. The allegations raised doubt about the DBST’s capability and knowledge of IE principles and practices.

It was established that some teachers had negative attitudes towards supporting learners or responding to their diverse needs. According to Mpu and Adu (2021), teachers’ attitudes are influenced by many factors, such as lack of support, their understanding of IE, lack of resources and lack of skills. Teachers’ attitudes might be influenced by their frustrations because of limited understanding of what constitutes IE and lack of support and resources. Some participants confirmed that they did not have the required skills, as they were not trained to overcome barriers to learning or to deal with diversity in their classrooms. This made it difficult for teachers to implement relevant intervention strategies to support learners who experience barriers to learning. It was further revealed that learners were left on their own, or teachers would only use those intervention strategies towards the end of the year, when they are supposed to finalise their learners’ progress reports.

The participants who worked in mainstream and full-service schools indicated that they had limited time for addressing diverse learning needs. Full-service and mainstream schools had overcrowded classrooms. Most participants from these schools indicated that they had 50 to 60 learners in their classrooms, which hindered them from assisting those learners who experience barriers to learning. Furthermore, most learners in their classrooms experienced learning difficulties. Overcrowded classrooms and a high learner-educator ratio can lead to negative teacher attitudes and didactical neglect (Meier & West 2020).

Didactical neglect includes the absence of differentiated learning. For example, participants indicated that despite their efforts to assist learners, they could not give individual attention to learners who experience barriers to learning, attesting to didactical neglect being present because of overcrowded classrooms.

Furthermore, some participants reported that they taught more than one subject in several classrooms, which made it difficult for them to respond to diverse learning needs. Most of the teachers who experienced challenges in responding to diverse learning needs and supporting learners taught in the intermediate phase. Like the finding by Meier and West (2020), our study showed that overcrowding of learners in classrooms not only caused barriers to accommodating diverse learning needs, it also led to a lack of discipline and disruptive classrooms.

Transportation was another reason stated for teachers not providing additional support to learners. Because most learners used school transport that departs immediately after school, participants could not devise a means of creating extra classes to assist those learners who experience barriers to learning.

The lack of parental involvement was another challenge. Some parents were in denial that their children experience barriers to learning, whereas others did not want to take the responsibility of working with teachers to support them. One of the reasons might be that parents were not aware of IE principles and the importance of their involvement in education to help their children.

### Limitations

Our study was conducted in one district, Bojanala, in the North West province, South Africa, and was limited to six schools, which included two special schools, two full-service schools and two mainstream schools. Consequently, the findings may lack generalisability to other schools and regions. Additionally, one special school, functioning as a resource centre, was notably well-resourced, with comprehensive support structures including school-based therapists, psychologists, nurses and learner support teachers who offered ongoing assistance to staff. Similarly, one of the full-service schools was also well-resourced and situated near to the special school, facilitating convenient access to its services. These unique resourcing conditions may not reflect the realities in other schools, particularly those in rural or under-resourced areas.

## Conclusion

Our study contributes to the knowledge on how IE can be implemented in such a way that it produces equitable and quality education for all learners. As indicated by the findings, issues of overcoming barriers to learning, responding to diverse learning needs and increasing the participation of all learners in the curriculum of educational institutions remain a challenge. The following recommendations are suggested:

It is important for the DBE to review the IE policies regarding the implementation of inclusion regularly and to ensure that these policies are advocated to all stakeholders. The DBE should ensure that the principles of IE, as envisaged in the policies, are implemented effectively in all education systems.

Successful implementation of IE requires collaboration and cooperation among all stakeholders, including the community, parents, teachers and various education sectors at district, provincial and national levels.

It is important that the DBE ensures equitable distribution of resources to all schools in response to diverse needs and support required to overcome barriers to learning. Special school resource centres should be accessible to enrol all learners with different disabilities because they are provided with all the appropriate resources.
